# Virtual reality for the rehabilitation of the upper limb motor function after stroke: a prospective controlled trial

**DOI:** 10.1186/1743-0003-10-85

**Published:** 2013-08-01

**Authors:** Andrea Turolla, Mauro Dam, Laura Ventura, Paolo Tonin, Michela Agostini, Carla Zucconi, Pawel Kiper, Annachiara Cagnin, Lamberto Piron

**Affiliations:** 1 I.R.C.C.S. Fondazione Ospedale San Camillo, Via Alberoni, 70-30126 Venezia-Lido, Italy; 2 Department of Statistics, University of Padova, Via Cesare Battisti, 241 – 35121 Padova, Italy; 3 Department of Neuroscience: SNPSRR, University of Padova, Via Giustiniani, 5 – 35128 Padova, Italy

**Keywords:** Stroke, Upper limb, Exercise therapy, Virtual reality, Motor recovery, Treatment outcome

## Abstract

**Background:**

Recent evidence has demonstrated the efficacy of Virtual Reality (VR) for stroke rehabilitation nonetheless its benefits and limitations in large population of patients have not yet been studied.

**Objectives:**

To evaluate the effectiveness of non-immersive VR treatment for the restoration of the upper limb motor function and its impact on the activities of daily living capacities in post-stroke patients.

**Methods:**

A pragmatic clinical trial was conducted among post-stroke patients admitted to our rehabilitation hospital. We enrolled 376 subjects who had a motor arm subscore on the Italian version of the National Institutes of Health Stroke Scale (It-NIHSS) between 1 and 3 and without severe neuropsychological impairments interfering with recovery. Patients were allocated to two treatments groups, receiving combined VR and upper limb conventional (ULC) therapy or ULC therapy alone. The treatment programs consisted of 2 hours of daily therapy, delivered 5 days per week, for 4 weeks. The outcome measures were the Fugl-Meyer Upper Extremity (F-M UE) and Functional Independence Measure (FIM) scales.

**Results:**

Both treatments significantly improved F-M UE and FIM scores, but the improvement obtained with VR rehabilitation was significantly greater than that achieved with ULC therapy alone. The estimated effect size of the minimal difference between groups in F-M UE and FIM scores was 2.5 ± 0.5 (P < 0.001) pts and 3.2 ± 1.2 (P = 0.007) pts, respectively.

**Conclusions:**

VR rehabilitation in post-stroke patients seems more effective than conventional interventions in restoring upper limb motor impairments and motor related functional abilities.

**Trial registration:**

Italian Ministry of Health IRCCS Research Programme 2590412

## Background

Stroke is a disorder associated with long term disability and is more common in older people [1]. The symptoms of stroke such as cognitive, motor and emotional sequalae often impact on a person’s level of independence and quality of life [2]. The purpose of neurological rehabilitation is to promote a rapid recovery from the manifold post-stroke deficits and the attainment of a lifestyle, as close as possible to the pre-morbid state [3].

A large body of evidence has demonstrated that the location of the stroke lesion is strictly related to the severity of motor function impairment affecting the upper limb, thus the involvement of deep anatomical structures (i.e. corona radiata, internal capsule) is related to poorer outcomes of motor function recovery [4,5]. A recent study reported that impairment of the upper limb motor function is present in more than 80% of all stroke patients, with 30% to 40% regaining some dexterity after six months [6]. Nevertheless, the upper limb remain not functional in performing activities of daily living (ADL) in up to 66% of all stroke patients [7], representing the most disabling of all the residual impairments.

New scientifically based treatments have considerably increased the repertoire of therapeutic and rehabilitative strategies. Such treatments include pharmacological interventions [8,9], constraint induced movement therapy [10], treadmill training with partial body weight support [11,12], robotic-assisted therapy [13], and Virtual Reality (VR) based interventions [14,15].

Studies in computational neuroscience have demonstrated that VR technology, providing enhanced feedback about movement characteristics, improved motor task learning and execution in healthy subjects, as compared with traditional training [16-19]. Exploiting these VR features, several authors have used VR based therapy aimed at relearning motor function in post-stroke patients. A recent Cochrane review [15], assessed the effect of VR treatment on recovery of motor, gait, balance, cognitive functions and ADL in stroke patients. The conclusion was that, despite encouraging results, strong evidence of better effects in favour of VR therapy compared to conventional therapy is still lacking. However, when considering only the upper limb treatment, all the studies included in the review indicated that the VR approach yielded better motor and functional outcomes than conventional therapy. Despite this assertion, the review identified the relatively small sample size of the studies included as one of the limitations for establishing stronger evidence.

The aim of this pragmatic clinical trial [20] was to further evaluate the effectiveness of VR based treatment, when provided in a routine hospital care setting, in restoring upper limb motor function and ADL capacities in a large number of post- stroke patients. Moreover, the influence of the severity of motor impairment and the distance between stroke onset and the start of the rehabilitation treatment (Stroke to Rehabilitation Interval – SRI) on motor and functional outcomes was also investigated.

## Material and methods

### Patients

The cohort of post-stroke patients considered for the study was selected from admissions to the Cerebrovascular Disease Unit of the San Camillo Hospital from 1998 and 2010. The Cerebrovascular Unit, according to Italian National Health System guidelines, has the potential to admit an average number of 100 inpatients/year, if they are judged to be likely to benefit from rehabilitation therapy. Within this cohort of patients, those suffering from hemiparesis due to a first stroke in the region of the middle cerebral artery (MCA) were screened for this study. Occlusion of the MCA frequently accompanied by contralateral hemiparesis is the most common type of lesion, occurring in the majority (65%) of strokes due to cerebrovascular diseases [21]. This patient group accounts for approximately 2/5 of the overall admitted patients. CT/MRI scan demonstrated different combinations of brain lesions, i.e. large damage involving most of the vascular territory of the MCA or more discrete lesions of the cortical and/or subcortical areas supplied by branches of the MCA. Moreover, the patients included in the study were those with a Motor Arm sub-score between 1 and 3 on the Italian version of the National Institutes of Health Stroke Scale (It-NIHSS) [22]. This score was considered as a reliable criterion for assessing the maintenance of residual voluntary motor activation.

The following conditions were considered as exclusion criteria: the presence of a moderate cognitive decline defined as a Mini Mental State Examination [23] score < 20/30 points; the finding of severe verbal comprehension deficit defined as a number of errors > 13 (Tau Points < 58/78) on the Token Test [24]; evidence of apraxia and neglect interfering with upper arm movements and manipulation of simple objects in all the directions within the visual field, as assessed through neurological examination and report in the patient’s clinical history or if there were evidence from neurological examination of behavioural disturbances (i.e. delusions, aggressiveness and severe apathy/depression) that could affect the compliance with the rehabilitation programs. Those criteria were decided for the feasible screening, within a defined population of stroke survivors, of those patients most likely capable of managing the interaction with a challenging rehabilitation setting, independently from their outcomes at baseline.

Most of the patients enrolled had already received previous rehabilitation interventions in the acute/subacute post-stroke period, according to the related guidelines of the Italian National Health System. Among the enrolled patients 129 (34.3%) were admitted to our hospital within the third month post-stroke to receive initial rehabilitation care, while 148 (39.4%) patients were admitted between the third month and the first year after stroke, having received previous rehabilitation treatment by a general rehabilitation unit. The remaining 99 (26.3%) patients enrolled were admitted at our unit after the first year after stroke: in this case they had already received an intensive rehabilitation care by a specialized rehabilitation unit different from ours.

Based on the previous evidence of efficacy of the VR systems [25-29], in our hospital the rehabilitation program for all post-stroke patients combined Upper Limb Conventional (ULC) and Reinforced Feedback in the Virtual Environment (RFVE) therapies. However, as the availability of the RFVE service was limited, some patients exceeding the waiting list were directed to receive additional ULC therapy. In accordance with this constraint, 263 patients were treated with ULC and RFVE therapies (RFVE group), and 113 patients received double sessions of ULC treatment (ULC group). In addition, the patients were further divided in subgroups based on: (1) the baseline severity of motor impairment detected with the F-M UE scale (mild: above 40/66; moderate: between 21 and 39; severe: ≤20/66); (2) the duration of SRI (between 1 and 3 months, between 4 and 12 months, exceeding 12 months).

The study was approved by the San Camillo Hospital Institutional Review Board and informed written consent was obtained by all participants at the time of enrolment.

### Interventions

All patients completed the rehabilitation programs, consisting of 40 sessions of daily therapy provided 5 days per week, for 4 weeks. The treatment protocol was delivered to inpatients in the case of missed sessions, lost treatments were rescheduled during hospitalization, in order to complete the assigned rehabilitation program.

In the RFVE group the daily treatment consisted of 1 hour of ULC therapy and 1 hour of VR therapy for the upper limb, while in the ULC group the patients underwent 2 hours of conventional treatment. In both intervention groups the physical therapists were constantly present during the session and modified the rehabilitation program in accordance with the patient’s current motor capacity and needs.

The equipment for VR therapy was the VRRS® (Virtual Reality Rehabilitation System. Khymeia Group. Noventa Padovana, Italy) and included a computer workstation connected to a 3D motion-tracking system (Polhemus Liberty™, Colchester, VT) and a high-resolution LCD projector displaying the virtual scenarios on a large wall screen. The VRRS® hardware was not substantially changed for the duration of the study and maintained the same level of performance throughout. The software only was upgraded in order to simplify the therapist’s interventions.

RFVE therapy involved performing different kinds of motor tasks with the patient holding a real manipulable object in their hands while interacting with a virtual scenario with movement monitored by means of a motion-tracking system.

**Figure 1:**
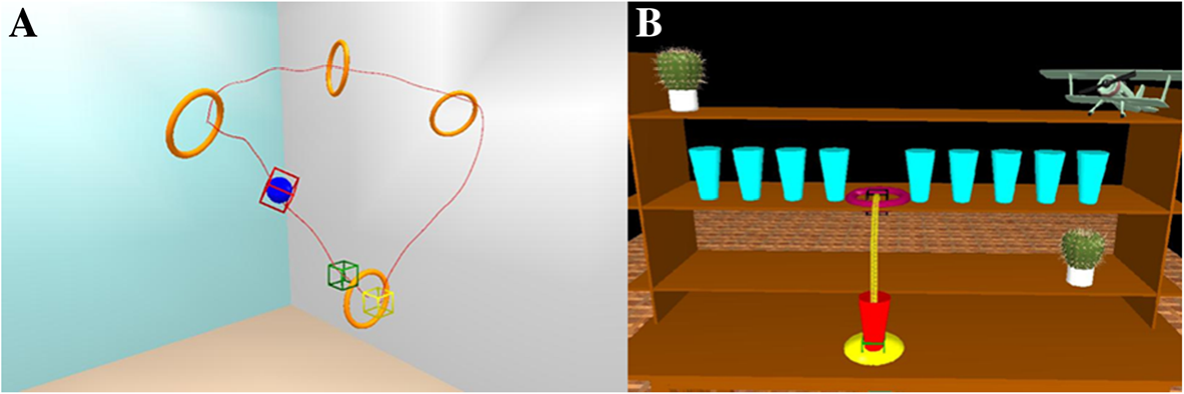
**Motor exercises in the virtual environment.** The two scenarios (VRRS® Khymeia Group, Ltd. Noventa Padovana. Italy) represent: **A)** a simple reaching movement: the patient has to raise the red glass and place it among the blue glasses on the shelf, according to a pre-recorded path (yellow line); **B)** a complex movement of increasing difficulty: the patient has to move the blue ball through the orange circles. The green box represents the start zone, while the yellow box represents the end zone to reach, following the circular-like displayed path.

For instance, a simple reaching-aiming movement, such as putting a glass on a shelf, is represented in the virtual scenario and was represented by a virtual glass and shelf. The physical therapist held in his hand a real glass with a receiver positioned on the object and performed the act of placing the glass on the virtual shelf. The virtual scenario displayed the correct movement path of the glass toward the shelf. The patient was then required to emulate the correct movement performed beforehand by the therapist. The correct trajectory was displayed in the background of the virtual scene to facilitate the patient’s perception and adjustment of his motion errors to target, by means of the on line visual knowledge of its performance and results (Figure 1A). The therapist selected the characteristics and complexity of the motor tasks by changing the position or orientation of the virtual objects. The complexity of the motor tasks could be enhanced by complicating the required movements adding objects/barriers into the virtual scenario. As a consequence, the patients were forced to activate different sets of upper arm muscles to perform the increasingly difficult task requirements (Figure 1B). The therapist was present at every session for the entire duration, as in a standard one-to-one setting. The therapist role was to manage the virtual environment to adapt it to the current patient’s physical condition and to guide the patient with verbal instructions in case of difficulties during the execution of the interactive exercise. At the end, the therapist discussed with the patients the results obtained during the therapy session.

The ULC program was based on traditional rehabilitation techniques aimed at restoring upper limb motor functions and based on the Bobath principles [30]. The patients were asked to perform a wide range of exercises, including: shoulder flexion-extension, abduction-adduction, internal-external rotation, circumduction, elbow flexion-extension, forearm pronation-supination, hand-digit motion. To facilitate motor skill relearning, patients underwent a sequence of motor tasks of increasing difficulty. Firstly, the patients were asked to control isolated movements without postural control and subsequently postural control was included. Later, complex motions were practiced. For example, patients were asked to follow with the arm simple or complex trajectories, to reach for different target positions, to grasp and manipulate objects. Based on residual motor capacities, the therapist could be placed next to the hemiparetic side of the patient, while seated on the edge of the therapy bed, for supporting the trunk as well as the arm and assisting them during the motor task execution. Conversely, in the case where the patient was able to control the trunk the therapist was seated in front of him controlling the correct execution of the task and providing verbal instructions to improve motor performance.

### Outcome measures

The Fugl-Meyer upper extremity (F-M UE) [31] and the Functional Independence Measure (FIM) [32] scales were chosen as outcome measures for the upper limb motor function and the independence in ADLs, respectively.

The hospital has independent clinical and rehabilitation facilities which are each supported by the research department for the development of clinical trials and implementation of translational findings. The interventions were carried out by rehabilitation staff while baseline and post-treatment assessments were carried out by clinical staff and therapists from the research department, all blind to the patient’s treatment allocation.

### Statistical analysis

Differences in demographic and clinical characteristics between ULC and RFVE groups at baseline were analysed using χ2 test, whereas mean age difference was analysed with a t–test. Wilcoxon and Mann–Whitney U tests were used to study the outcome differences within and between ULC and RFVE treatment groups. In addition, a generalised linear model (GLM) with gamma identity link function, was used to study whether F-M UE and FIM scores were significantly different post treatment, between experimental and control groups, as well as in the subgroups with different severity of motor impairment and progressively longer SRI. The demographic and clinical variables at baseline in the two groups were used as continuous covariates [33,34].

All the statistical analyses were performed using the free software R [35] and statistical significance was set at P ≤ 0.05.

## Results

Table 1 shows that the number of patients included in the study was higher in the RFVE group (70% of the subjects) than in ULC group (30% of the subjects). Nevertheless, the percentages of the RFVE and ULC patients were comparable on demographic and clinical features. As the only exception, the patients in the RFVE group were on average 4 years younger than the ULC therapy group (P ≤ 0.001). As depicted in Table 2, baseline F-M UE scores were comparable in both patient groups and in the subgroups defined by different severity of motor impairments and by progressively longer SRI. At the end of the treatment, there was a significant increase in the F-M UE score of 4% (P < 0.001) in the ULC group and 10% (P < 0.001) in the RFVE group. Comparison of the two groups also revealed significantly greater motor improvement with the RFVE therapy (P < 0.001).

**Table 1:** Demographic and clinical characteristics of the patients

**Patients**		**ULC therapy (n=113)**	**RFVE therapy (n=263)**	**p-value**
Sex (M/F)		72/41 (64%/36%)	157/106 (60%/40%)	0.5 ^a^
Age (years ± SD)		65.4 ± 12.5	60.2 ± 14.3	<0.001 ^b^
Ischemic/hemorrhagic stroke		82/31 (73%/27%)	188/75 (71%/29%)	0.6 ^a^
Affected hemisphere (R/L)		58/55 (51%/49%)	126/137 (48%/51%)	0.7 ^a^
Upper limb motor impairment	Severe (F-M UE≤20)	19 (17%)	35 (13%)	0.6 ^a^
	Moderate (21≤F-M UE≤40)	29 (26%)	72 (27%)	0.9 ^a^
	Mild (F-M UE>40)	65 (57%)	156 (59%)	0.8 ^a^
Stroke to Rehabilitation Interval	≤ 3 months	32 (28%)	68 (26%)	0.7 ^a^
	3 < months < 12	57 (50%)	113 (43%)	0.2 ^a^
	≥ 12 months	24 (21%)	82 (31%)	0.1 ^a^

Values are expressed as numbers and percentages. *ULC* Upper Limb Conventional, *RFVE* Reinforced Feedback in Virtual Environment, *F-M UE* Fugl-Meyer Upper Extremity.

^a^ χ2 test.

^b^ t-test.

**Table 2:** Effect of therapies on Fugl-Meyer Upper Extremity scale

			**ULC therapy**			**RFVE therapy**	
		**n**	**Before**	**After**	**n**	**Before**	**After**
Overall		113	41.1 ± 17.6	44.1 ± 17.3*	263	41.7 ± 16.1	48.2 ± 15.2*†
Motor impairment	F-M UE score ≤ 20	19	11.1 ± 6.5	14.6 ± 8.5*	35	11.8 ± 5.2	19.0 ± 9.0*†
	21 ≤ F-M UE score ≤ 40	29	32.5 ± 5.5	36.1 ± 6.8*	72	32.2 ± 5.9	41.5 ± 7.6*†
	F-M UE score > 40	65	53.8 ± 7.1	55.9 ± 8.1*	156	52.7 ± 7.3	57.8 ± 6.4*†
Stroke to Rehabilitation Interval	≤ 3months	32	47.7 ±16.4	51.3 ± 15.2*	68	44.9 ± 14.0	53.8 ± 11.5*†
	3 < months < 12	57	38.9 ± 17.5	41.7 ± 17.2*	113	39.5 ± 17.2	46.0 ± 16.0*†
	≥ 12 months	24	37.7 ± 17.7	40.3 ± 18.0*	82	41.8 ± 15.9	46.7 ± 15.7*

Data are presented as mean ± SD. *n* number of patients, *ULC* Upper Limb Conventional, *RFVE* Reinforced Feedback in Virtual Environment, *F-M UE* Fugl – Meyer Upper Extremity.

* p ≤ 0.05 within group analysis (Wilcoxon test).

† p ≤ 0.05 between group analysis (Mann–Whitney U test).

In the subgroup analysis, patients with severe, moderate and mild motor impairment, showed a significant improvement in F-M UE scores by 5%, 5% and 3% respectively after ULC therapy, and by 11%, 14% and 8% respectively after RFVE treatment. All the above improvements were significantly higher in the RFVE than in the ULC treated patients (P < 0.001) (Table 2).

As for the SRI subgroups, ULC therapy significantly improved F-M UE scores by 4%, in all subgroups, while RFVE treatment significantly improved F-M UE scores by 13%, 10% and 7% in the subjects with SRI lower than 3 months, between 3 and 12 months and above 12 months, respectively. The changes in F-M UE scores were significantly higher after RFVE than in the ULC treatment only in the groups with an SRI up to 12 months.

Table 3 shows that baseline FIM scores were 5% to 7% higher in the RFVE group and subgroups, compared to ULC. Only the patient subgroups with a SRI longer than 3 months yielded a significantly different baseline FIM values between the two treatment groups (between 3 and 12 months, P= 0.022; months > 12, P=0.007).

**Table 3:** Effect of therapies on Functional Independence Measure scale

			**ULC therapy**			**RFVE therapy**	
		**n**	**Before**	**After**	**n**	**Before**	**After**
Overall		113	95.0 ± 21.4	101.9 ± 19.1*	263	103.2 ± 20.7	110.8 ± 16.4*
Motor impairment	F-M UE score ≤ 20	19	84.2 ± 26.2	89.3 ± 24.2*	35	92.8 ± 20.7	101.2 ± 17.9*
	21 ≤ F-M UE score ≤ 40	29	94.0 ± 15.5	102.0 ± 15.9*	72	100.4 ± 24.0	108.0 ± 19.0*
	F-M UE score > 40	65	98.5 ± 21.3	105.6 ± 17.3*	156	106.9 ± 18.0	114.2 ± 13.5*
Stroke to Rehabilitation Interval	≤ 3months	32	88.2 ± 25.8	100.0 ± 22.6*	68	95.9 ± 23.3	110.1 ± 16.5*
	3 < months < 12	57	95.6 ± 18.2	100.4 ± 16.3*	113	101.7 ± 20.0†	108.2 ± 17.2*
	≥ 12 months	24	102.5 ± 20.2	108.3 ± 20.0	82	111.0 ± 16.8†	114.8 ± 14.4*

Data are presented as mean ± SD. *n* number of patients *ULC* Upper Limb Conventional, *RFVE* Reinforced Feedback in Virtual Environment, *F-M UE* Fugl – Meyer Upper Extremity.

* p ≤ 0.05 within group analysis (Wilcoxon test).

† p ≤ 0.05 between group analysis (Mann–Whitney U test).

After therapy, within group comparisons demonstrated that both therapies significantly improved the FIM score, and those effects were similar across the two treatments. However, given the different baseline FIM scores between ULC and RFVE groups, we performed additional analysis for a more reliable comparison between groups. Therefore a GLM regression analysis with gamma identity link function was performed in order to evaluate: (1) whether the differences between groups observed in the final F-M UE and FIM scores were related to any covariation with their baseline values; (2) the minimal different effect on the F-M UE and FIM scores, due to each treatment independent of demographic and clinical characteristics of the patients.

The assumption for applying this type of regression model is based on the asymmetric distribution of the values based on the box-plots of the F-M UE and FIM scores in the RFVE and ULC groups [see Additional file 1].

**Figure 2:**
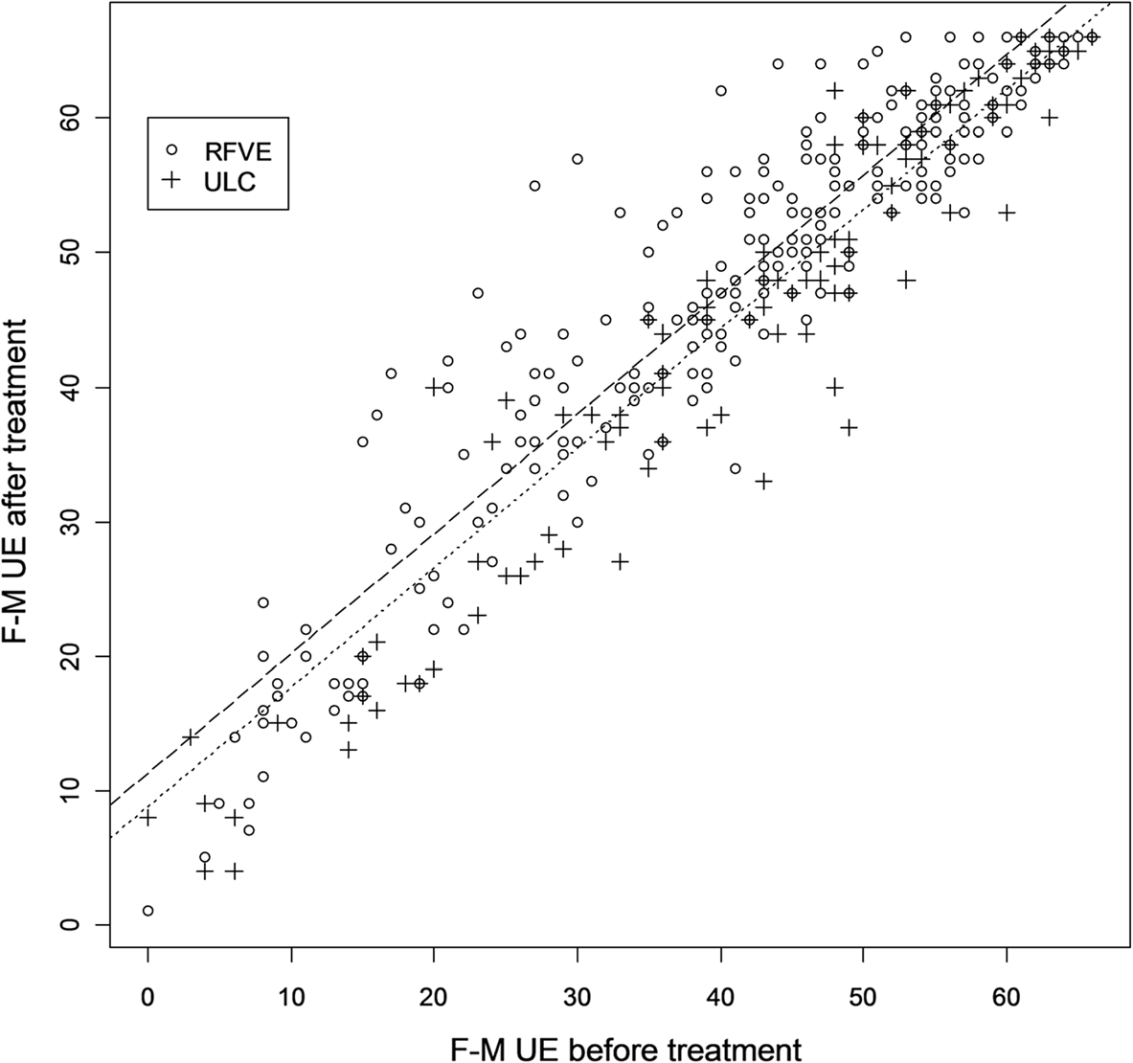
**Scatter plot of the Fugl - Meyer Upper Extremity scores before and after treatment.** Fitted models in the RFVE and ULC groups are displayed in dashed and dotted line, respectively. The plot shows the better score at F-M UE scale after RFVE compared with ULC therapy, adjusted for the severity of pre treatment motor impairment. Empty circle (о) represents individual F-M UE score of the RFVE patients group, cross symbol (+) represents individual F-M UE score of the ULC patients group.

The GLM analysis showed that the F-M UE scores were significantly higher in the overall RFVE than in the ULC group post-treatment (effect size: 2.5 ± 0.5 pts, P<0.001) (Figure 2). Similar results were seen in the analysis of the severe and moderately impaired subgroups (effect size severe: 2.5 ± 0.5 pts, P<0.001; effect size moderate: 4.4 ± 1.3 pts, P<0.001), however the effect was not significant in the mild subgroup (effect size mild: 2.5 ± 2.0 pts, P=0.21). F-M UE scores were also significantly higher in the RFVE in all the three SRI subgroups (effect size ≤3mo: 2.5 ± 0.5 pts, P<0.001; effect size 3-12mo: 4.9 ± 0.9 pts, P<0.001; effect size ≥12mo: 4.6 ± 0.9 pts, P<0.001).

**Figure 3:**
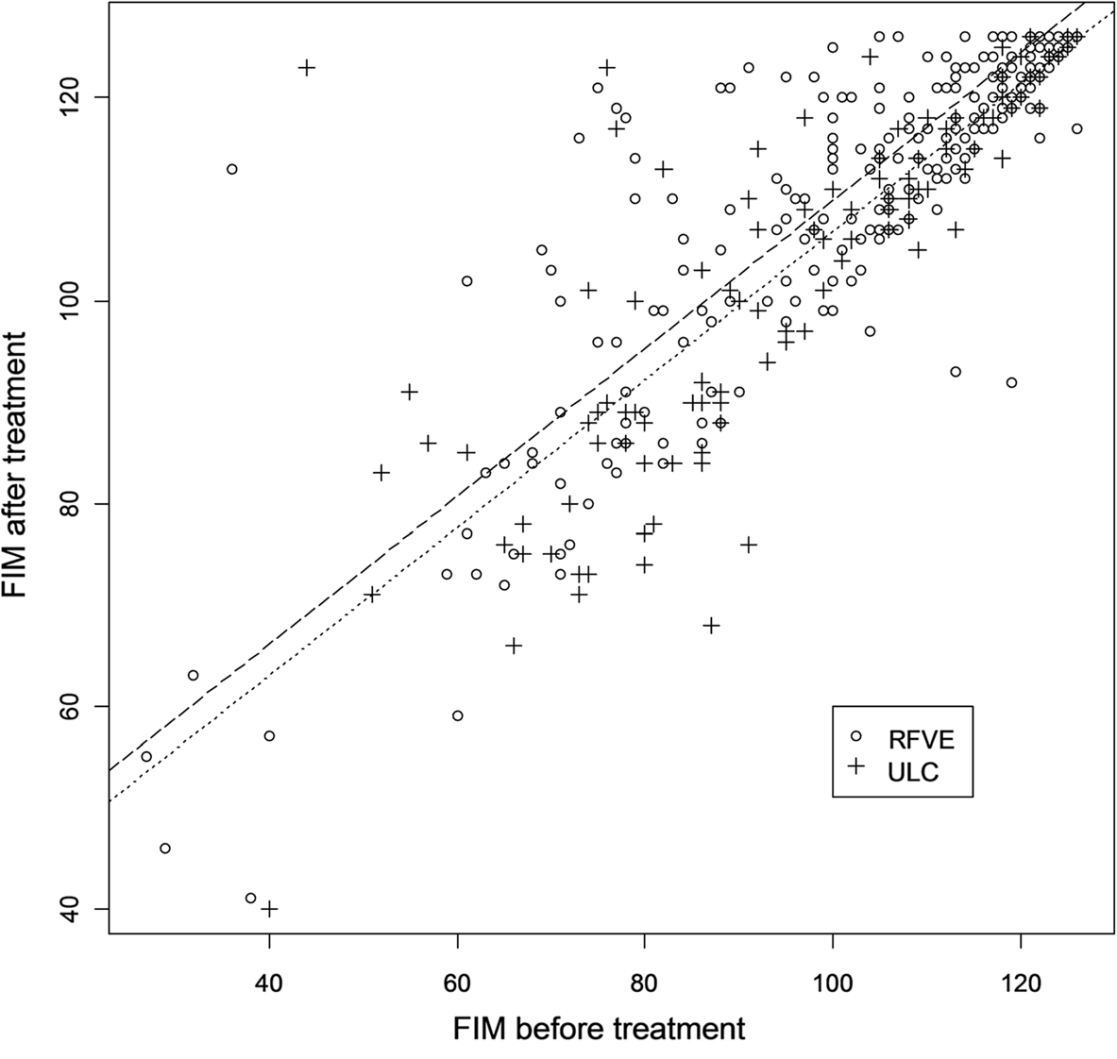
**Scatter plot of the Functional Independence Measure scores before and after treatment.** Fitted models in the RFVE and ULC groups are displayed in dashed and dotted line, respectively. The plot shows the better score at FIM scale after RFVE compared with ULC therapy, adjusted for the severity of pre treatment motor impairment. Empty circle (о) represents individual FIM score of the RFVE patients group, cross symbol (+) represents individual FIM score of the UCL patients group.

Similarly, the FIM scores were significantly higher in the overall RFVE than in the ULC group post-treatment (effect size: 3.2 ± 1.2 pts, P=0.007) (Figure 3). We also observed that FIM scores were significantly higher after RFVE than in ULC in all the three subgroups with different degrees of motor impairment (effect size severe: 3.2 ± 1.2 pts, P=0.007; effect size moderate: 3.3 ± 1.7 pts, P=0.04; effect size mild: 4.7 ± 1.6 pts, P=0.003), as well as in the three SRI subgroups (effect size ≤3mo: 3.2 ± 1.2 pts, P=0.007; effect size 3-12mo: 6.4 ± 1.4 pts, P<0.001; effect size ≥12mo: 6.4 ± 1.6 pts, P<0.001).

In both groups, none of the baseline clinical and demographic variables interfered significantly with the F-M UE and FIM post-treatment results. This was also true for age (F-M UE: P=0.52; FIM: P=0.11), even though the mean age of both groups was significantly different.

Summarizing the above results, the GLM regression analysis showed that RFVE therapy yielded significantly better post-treatment F-M UE and FIM improvements than ULC therapy. Moreover, these findings were independent of baseline patients’ clinical and demographic characteristics.

## Discussion

To our knowledge this is the largest trial, available in the literature, evaluating the effectiveness of post-stroke VR-based rehabilitative intervention, in a routine care setting. Our results are in agreement with those of the seven trials including 205 participants selected in the Cochrane review [15] and suggest that VR intervention combined with UCL treatment is more effective than conventional therapy alone in restoring upper limb motor function and ADLs, in the aftermath of stroke. As expected, the effects of combined RFVE and ULC therapies were more remarkable on F-M UE than on FIM total score. This is not surprising since the treatment is aimed at restoring upper limb motility, which in turn may improve only FIM sub-items related to motor skills, not those concerning communication, socialisation and sphincter control.

The rationale underpinning the effects of the VR application is based on the commonly recognised fact that learning new motor tasks is dependent on the feedback derived from the performance of the task itself. In healthy subjects, VR systems improve the effect of motor exercises learning and execution by increasing the available feedbacks, as compared with conventional training programs [16-19]. As a consequence, it is conceivable that the exploitation of the augmented feedbacks, such as knowledge of performance and of results, can facilitate the reacquisition of the compromised motor abilities. Several other features of VR systems may be relevant in neurological rehabilitation. Grading the intensity and the difficulty of the motor task in individual patients may stimulate more effective mechanisms of brain reorganisation involved in restoring anatomical/motor deficits [36]. In addition, the possibility of modifying the features of the virtual scenario makes the rehabilitation sessions more attractive and pleasant. In this context, patients are continuously challenged by newly designed tasks, which implies a more active participation in the requested exercises, potentially improving the outcomes and hastening the recovery process. With time, many patients learn how to handle the VR equipment with little supervision by the physical therapists. It should be acknowledged that several equipments developed for VR applications in rehabilitation are currently available. Due to their technological content variety exists through different systems, thus the results from our study may not necessarily apply to different VR equipments. In particular, caution should be taken when considering low cost commercially available gaming systems as ready to use devices for providing motor rehabilitation therapy based on VR. In our opinion the opportunity of disseminating rehabilitation therapy based on VR approaches for regaining motor function may be of paramount relevance to both post-stroke subjects and to the Health System since the former may exploit the effects of prolonged rehabilitation sessions, sparing the available staffing resources.

Some limitations are present in the current study and needed to be acknowledged. This pragmatic trial was aimed at comparing the effect of the RFVE therapy with standard treatment in a real clinical setting, thus a non randomised technique was used for patients’ allocation. This approach makes it difficult the detection of possible confounding factors biasing our findings. For this reason, the major stroke prognostic factors were modelled to estimate their weight on the observed results, but due to the chosen methodology is not possible to exclude other unknown factors influencing our findings. The long time of recruitment could be considered as a further limitation of the study. Nevertheless, throughout the ten years enrolment screening, assessment and treatment protocols didn’t change and the background technologies were updated only to follow commercial advancement. Increasing evidence supports the use of virtual reality for stroke rehabilitation, but small studies are currently affecting the possibility to reach a conclusive agreement within the neurorehabilitation community. Based on this need, several authors are encouraging the designing of large studies characterised by robust outcomes and adequate power and this trial is the first one considering an open population of real stroke inpatients. As a consequence future directions for research should aim at applying a similar controlled therapy in a large randomised clinical trial.

## Conclusion

In this study we prospectively evaluated the therapeutic effectiveness of combined RFVE and ULC therapy in comparison with ULC therapy alone in two large groups of patients affected by impairments of the upper arm motor function and of ADL capacities, due to a stroke in the MCA territory. The patient groups were well balanced with respect to gender, type of stroke, affected hemisphere, severity of motor impairments and SRI. Although the mean age of RFVE was significantly lower compared with the ULC group, the 4 year difference did not affect the outcomes. All patients included in the ULC and RFVE therapy group completed their assigned rehabilitation program. None of the VR treated subjects complained of visual disturbances, nausea, headache or other discomforts, usually reported in relation to the use of immersive VR equipments [37]. At the end of the rehabilitation program, both therapies significantly increased F-M UE and FIM scores, in comparison to baseline values, in the overall group of post-stroke patients and in the subgroups with different severity of motor impairments and SRI. The observed improvements were significantly higher in the RFVE treated group than in the ULC treated patients.

In the aftermath of stroke, the association of VR based rehabilitation with traditional restorative approaches improves the effectiveness of restoring upper limb motor function and ADL capacities, compared with conventional rehabilitation care alone. The above findings, in agreement with previous evidence, suggest that VR treatments represent a valuable therapeutic option and should be more widely considered in rehabilitation programs designed for post-stroke patients.

## Abbreviations

VR: Virtual reality; ULC: Upper limb conventional; F-M UE: Fugl-Meyer Upper Extremity; FIM: Functional Independence Measure; ADL: Activity of daily living; SRI: Stroke to rehabilitation interval; MCA: Middle cerebral artery; RFVE: Reinforced feedback in virtual environment; VRRS®: Virtual reality rehabilitation system; GLM: Generalized linear model; IT – NIHSS: Italian version of the National Institutes of Health Stroke Scale.

## Competing interests

The author(s) declare that they have no competing interests.

## Authors’ contributions

AT, MD, LV, LP, AC were involved in analysis and interpretation of data, as well as in drafting and revising the manuscript, PT revised it critically for important intellectual content. AT, LP, MD, PT, MA, CZ made substantial contributions to conception and design of the study. AT, MA, CZ, PK were involved in acquisition of data. All authors read and approved the final manuscript.

## Supplementary Material

Additional file 1 Study of the outcomes’ distribution for the GLM analysis. Click here for file
