# Supplementary material for: Virtual reality for the rehabilitation of the upper limb motor function after stroke: a prospective controlled trial
Source: J Neuroeng Rehabil. 2013 Aug 1;10:85. doi: 10.1186/1743-0003-10-85 (PMC3734026; doi:10.1186/1743-0003-10-85)
Supplement: Additional file 1 — Study of the outcomes’ distribution for the GLM analysis. [file 1743-0003-10-85-S1.pdf]

ADDITIONAL FILE 1

<u>TITLE</u>

Study of the outcomes' distribution for the GLM analysis.

<u>Supplemental Methods</u>

To study the empirical distribution in every subgroup, as defined in the patients section, were displayed the box-plots of the FIM and F-M UE scores before and after the treatment, in the RFVE and UCL groups, according to the levels of motor impairment and SRI (Figure S1a and Figure S1b, respectively). From the visual analysis the plots suggested that the differences in both outcomes scores may depend on level of motor impairment and SRI with a strongly asymmetric empirical distributions of both FIM and F-M UE measurements. This feature support the use of the class of the general linear model (GLM) with identity link function [1], to study the effects of the RFVE and UCL therapies on FIM and F-M UE outcomes.

<u>Supplemental Figure</u>

S1. Distribution of the clinical outcomes values according to the motor impairment and stroke rehabilitation interval subgroups.

a) Outcomes' distribution according to the levels of motor impairment.

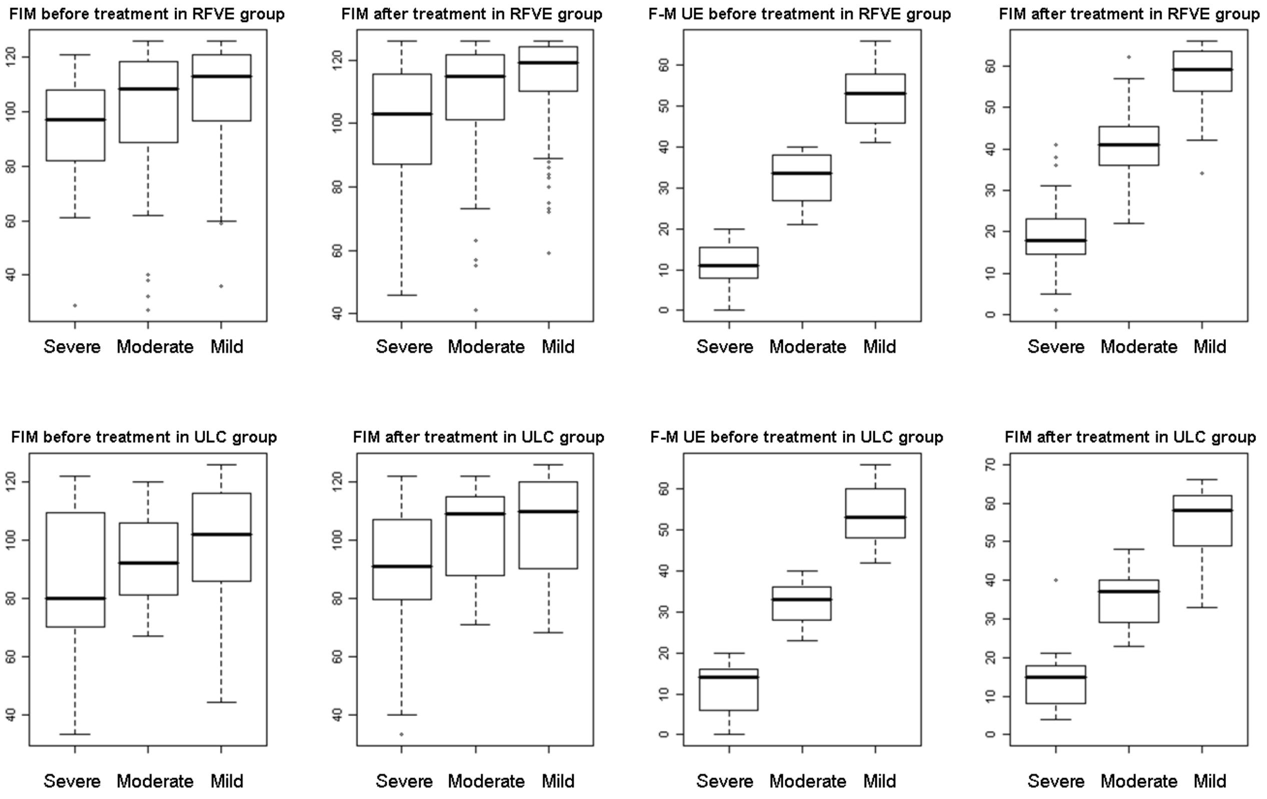

FIM = Functional Independence Measure, F-M UE = Fugl-Meyer Upper Extremity, RFVE = Reinforced Feedback in Virtual Environment, ULC = Upper Limb Conventional.

b) Outcomes’ distribution according to the stroke rehabilitation interval.

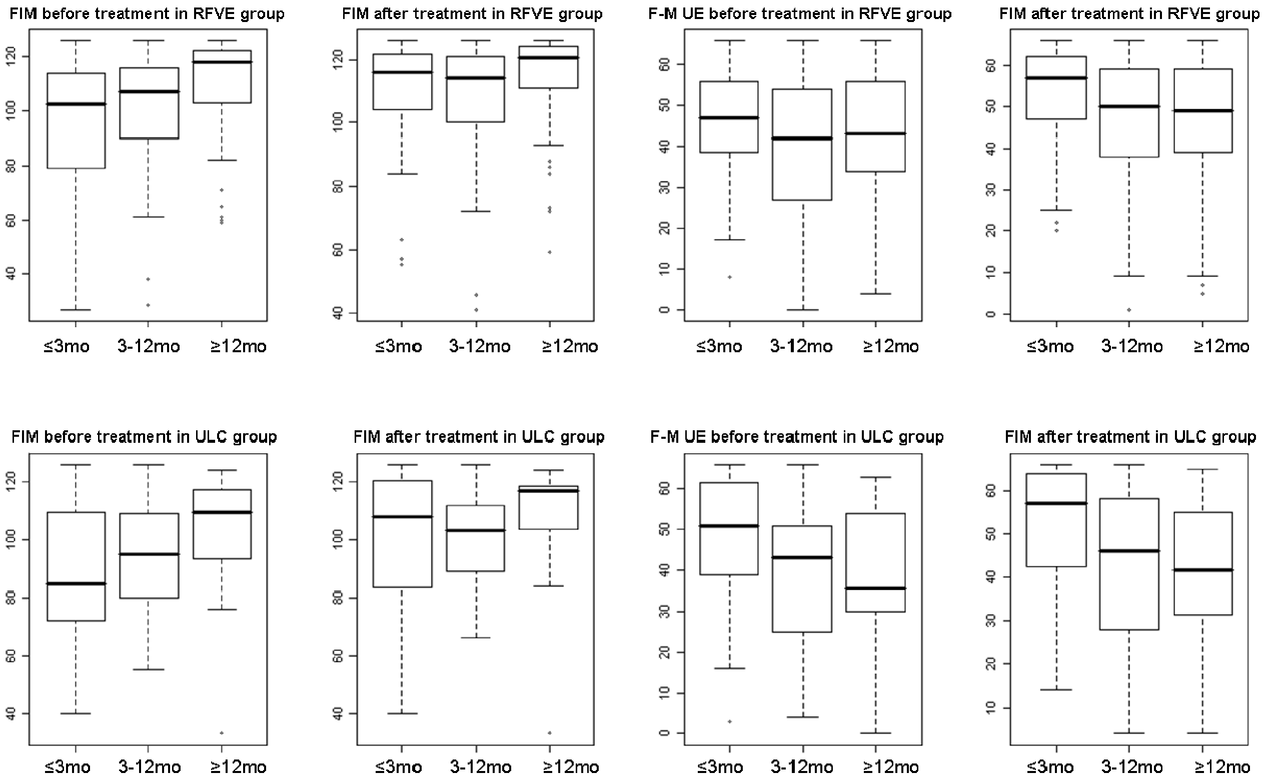

FIM = Functional Independence Measure, F-M UE = Fugl-Meyer Upper Extremity, RFVE = Reinforced Feedback in Virtual Environment, ULC = Upper Limb Conventional.

<u>Supplemental Reference</u>

1. Venables WN, Ripley BD. Modern Applied Statistics with S. 4th Ed. New York, NY: Springer; 2002.
